# Computational and Population-Based HLA Permissiveness to HIV Drug Resistance-Associated Mutations

**DOI:** 10.3390/pathogens14030207

**Published:** 2025-02-20

**Authors:** Rizwan Mahmud, Zoë Krullaars, Jolieke van Osch, David Rickett, Zabrina L. Brumme, Kathryn S. Hensley, Casper Rokx, Rob A. Gruters, Jeroen J. A. van Kampen, Thibault Mesplède

**Affiliations:** 1 Viroscience Department, Erasmus University Medical Center, 3015GD Rotterdam, The Netherlands; nayeemgebdu@gmail.com (R.M.); z.krullaars@erasmusmc.nl (Z.K.); j.vanosch@erasmusmc.nl (J.v.O.); r.gruters@erasmusmc.nl (R.A.G.); j.vankampen@erasmusmc.nl (J.J.A.v.K.); 2 British Columbia Centre for Excellence in HIV/AIDS, Vancouver, BC V6Z1Y6, Canada; drickett@bccfe.ca (D.R.); zbrumme@bccfe.ca (Z.L.B.); 3 Faculty of Health Sciences, Simon Fraser University, Burnaby, BC V5A1S6, Canada; 4 Departments of Internal Medicine (Infectious Diseases) and Medical Microbiology and Infectious Diseases, Erasmus University Medical Center, 3015GD Rotterdam, The Netherlands; k.hensley@erasmusmc.nl (K.S.H.); c.rokx@erasmusmc.nl (C.R.)

**Keywords:** HIV, HLA, antiretroviral drug resistance, antigen binding, cytotoxic T-lymphocytes, HLA-B57

## Abstract

The presentation of HIV peptides by the human leukocyte antigen (HLA) complex to CD8+ cytotoxic T-cells (CTLs) is critical to limit viral pathogenesis. HIV can mutate to evade HLA-restricted CTL responses and resist antiretroviral drugs, raising questions about how it balances these evolutionary pressures. Here, we used a computational approach to assess how drug resistance-associated mutations (RAMs) affect the binding of HIV-1 subtype B or C peptides to the most prevalent HLA alleles in US, European, and South African populations. We predict RAMs that may be favored in certain populations and report the under-representation of Y181C in people expressing HLA-B*57:01. This finding agreed with our computational predictions when Y181C was at the major anchor site P2, suggesting the potential relevance of our approach. Overall, our findings lay out a conceptual framework to study the implications of HLA alleles on the emergence of HIV RAMs at the individual and population levels.

## 1. Introduction

Nearly 40 million people worldwide, including 1.8 million children, currently live with the human immunodeficiency virus (HIV) (UNAIDS: https://www.unaids.org/en/resources/fact-sheet accessed on 18 February 2025). This rapidly mutating retrovirus infects CD4+ T-cells, macrophages, and dendritic cells, progressively damaging the immune system and enabling the development of life-threatening opportunistic infections. Lifelong treatment with antiretroviral therapy (ART) commonly permits the restoration of CD4+ T-cell counts and immune function, but latent infection in retroviral reservoirs prevents a cure. Even in the setting of an optimally treated HIV infection by antiretroviral therapy, a substantially increased risk of comorbidities remains compared to people without HIV [1]. In addition to antiretroviral drugs, the immune system, including CD8+ T-cells, contributes to limiting HIV pathogenesis by targeting infected cells [2].

CD8+ T-cells recognize HIV-infected cells by binding human leukocyte antigen (HLA) class I molecules that present viral antigens on the cell surface. This recognition allows CD8+ cytotoxic T-lymphocytes (CTLs) to target infected cells via secreted molecules, such as perforin or granzyme B, or through the activation of the Fas/FasL pathway [3]. Additionally, the HLA-CD8+ T-cell axis plays a significant role in HIV control, not only by directly targeting infected cells but also by activating B-cells to produce neutralizing antibodies [4,5]. Elite HIV-1 controllers with specific HLA alleles can control HIV replication without needing ART [6,7,8]. Amongst protective HLA alleles, HLA-B27 and HLA-B57 are the best characterized [9,10,11]. Reciprocally, some HLA alleles are associated with faster disease progression [12].

HIV escapes immune recognition primarily through the development of mutations, with some of the best-characterized examples occurring in the immunodominant *gag* region of the viral genome [13,14,15]. Also, suboptimal ART can drive the emergence of resistance-associated mutations (RAMs), which confer resistance to specific drugs or drug classes and are closely monitored in case of virological failure.

However, the evolution of both RAMs and immune escape mutations can be constrained by the reduced viral fitness associated with certain mutations [13,16,17,18,19,20,21,22].

The interplay between RAMs and HLA alleles is well documented, as discussed in detail below, with some specific alleles favoring some mutations, while others limit the emergence of specific RAMs [23,24,25,26,27,28,29,30,31,32,33,34].

In this paper, we used a computational- and population-based approach to systemically investigate the effects of RAMs on HLA binding. We observed that RAMs could decrease, increase, or have a neutral effect on HLA binding. We refer to the scenario where a RAM increased HLA binding as “non-permissiveness”, because enhanced presentation of RAM-containing peptides on the surface of infected cells may theoretically increase their recognition and targeting by CTLs compared to cells presenting WT peptides. This could limit the emergence of specific RAMs in people expressing certain HLA alleles. Conversely, we defined “permissiveness” as the situation where a RAM reduced HLA binding, thus potentially facilitating CTL escape and accelerating resistance development by simultaneously enabling drug resistance and immune escape.

## 2. Materials and Methods

### 2.1. Cohort and Ethics Statement

The British Columbia Centre for Excellence in HIV/AIDS (BC-CfE) is a provincially funded agency that is responsible for providing treatment to all people with HIV (PWH) in the province of British Columbia, Canada. As part of its mandate, the BC-CfE maintains the HIV Drug Treatment Program (DTP) clinical registry that captures HIV-related clinical information from all PWH in BC, including HIV drug resistance genotypes and HLA-B*57:01 genotypes (collected for abacavir hypersensitivity screening). The analysis of the relationship between HLA-B*57:01 allele and HIV RAM carriage in the BC-CfE DTP clinical registry was approved by the Providence Health Care/University of British Columbia and Simon Fraser University Research Ethics Boards under protocols H08-00962 (most recent approval date 27 December 2024) and H04-50276 (most recent approval date 27 December 2024). All participants provided written informed consent.

### 2.2. Selection of Drug Resistance-Associated Mutations

Major RAMs were selected from the Stanford University HIV Drug Resistance Database version 9.2, accessed in 2022 [35,36,37]. These RAMs were identified as “major” by the Stanford Drug Resistance Database team based on their common clinical detection. The substitutions target four drug classes: integrase strand transfer (INSTI), nucleos(t)ide reverse transcriptase (NRTI), non-nucleoside reverse transcriptase (NNRTI), and protease inhibitors (PI). In total, 85 substitutions in reverse transcriptase, protease, and integrase (Table 1) were analyzed. Insertions (e.g., T69) were excluded because of the difficulties of preparing the peptides for analysis. RAMs against entry inhibitors (not included in the Stanford Drug Resistance Database) and lenacapavir, a capsid inhibitor, were not included in this analysis.

### 2.3. Selection of HLA Alleles

First, HLA alleles were selected based on their published ability to recognize specific HIV peptides, as compiled on the HIV Database of the Los Alamos National Laboratory (https://www.hiv.lanl.gov/content/immunology/index.html, accessed on 16 February 2022). Additional HLA alleles were selected based on their high frequency (>5%) in the European [38] and American population. The latter was further divided into Americans of European [39] and African descent [40]. This sub-analysis was performed because race is part of insurance-collected data in the U.S.A. that may provide testable hypotheses regarding the relevance of our work. Indeed, the emergence rates of specific RAMs may be compared between people of various race, granted that they use the same treatment regimen and have comparable clinical parameters. Selected alleles are listed in Table 2. Alleles commonly found in the South African population have been previously published [41].

### 2.4. HLA-Peptide Binding Prediction

Peptide presentation by various HLA alleles was predicted using the NetMHCpan 4.0 tool hosted by the Technical University of Denmark (https://services.healthtech.dtu.dk/service.php?NetMHCpan-4.0, accessed 8 March 2022), which uses artificial neural networks to perform its calculation [42,43,44]. When we initiated this project, NetMHCpan v.4.1 was not yet available. Partial validation using the updated version 4.1 was performed. We also validated some of our results with another prediction tool called MHCseqNet [45]. Overall, NetMHCpan was chosen because of its training on a very large combination of binding data, input flexibility, recommendation by the Immune Epitope Database, and its ability to predict binding for any MHC class I allele [44,46,47].

We performed three different analyses. First, we used the CTL/CD8+ epitope map of the HIV Immunology Database at the Los Alamos National Laboratory and quantified the effect of RAMs on the binding of published HLA-specific peptides (https://www.hiv.lanl.gov/content/immunology/maps/ctl/ctl.pdf, accessed 16 February 2022). Fold changes in binding scores were calculated as the RAM:WT peptide binding score ratio. Since a higher binding score indicates a better HLA presentation, an FC > 1 served to identify non-permissiveness, whereas an FC < 1 indicated permissiveness. Specifically, an FC > 1 indicates an increase in binding for the mutated peptides compared to their WT counterparts. A clinically relevant cut-off could not be defined. An FC = 1 indicated that the RAM did not change the ability of the peptides to bind the HLA allele (neutral). Illustrations and statistical analyses were created with Prism v.9.5.0 (GraphPad Software, LLC, San Diego, CA, USA).

Second, for the population-based analyses, frequent HLA alleles (Table 2) were screened against all 85 RAMs (Table 1). In this case, though HLA class I alleles can typically present 8–11 amino acid-long peptides, we restricted our analyses to 9-mer peptides, as this is the most common peptide length [48]. Accordingly, the input peptides were 17-mers, with 8 amino acids surrounding the amino acid of interest (X_8_-X_RAM_-X_8_). The surrounding amino acids were derived from consensus amino acid sequences of subtype B or C. Specifically, HXB2 (GenBank: K03455.1) was used to generate background amino acid sequences surrounding the RAMs for subtype B. The Los Alamos National HIV Library subtype C consensus (120CG1) was used to generate background amino acid sequences for this subtype. From the 17-mers, NetMHCpan 4.0 processed all 9-mer peptides spanning the position of interest, yielded binding scores per HLA allele for each 9-mer, and identified weak (>2% rank) and strong (>0.5% rank) HLA-binding peptides. These were standard thresholds for NetMHCpan predictions. Peptides that were neither weak nor strong binders were excluded from downstream analyses. Notably, in some instances, 9-mers predicted to bind significantly (weak or strong binders) were different in the WT and RAM peptides. In such cases, the score of the corresponding other 9-mer peptide was used to calculate the FC, even if it did not qualify as a weak or strong binder. FC scores for all HLA-restricted peptides containing a RAM and predicted to bind (weak or strong binders) were averaged, log-transformed, and summarized in heatmaps (Prism v.9.5.0) or a table.

Third, we performed a detailed analysis focusing on HLA-B57:01 and the Y181C substitution, guided by an analysis of a large dataset of HIV drug resistance genotypes paired with HLA-B*57:01 genotypes (see below). This analysis included all binding scores (not limited to weak or strong binders) and 9–11-mers. Exclusively for this analysis, binding affinity was used rather than scoring values.

## 3. Results

### 3.1. Drug Resistance-Associated Mutations Change the Binding of HIV-1 Subtype B Peptides to HLA Alleles

**Figure 1:**
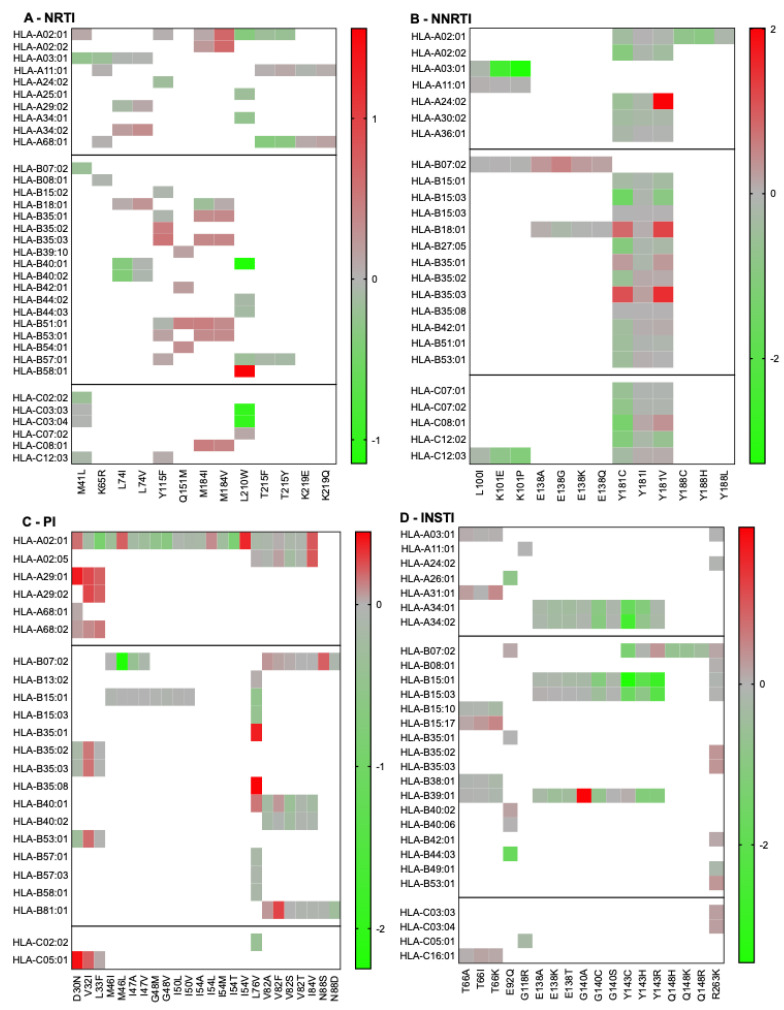
Permissiveness and non-permissiveness heatmaps of published RAMs/HLA alleles combinations. Log-transformed fold changes in binding are color-coded. Red indicates non-permissiveness, green permissiveness, and grey neutrality. Since the peptides included in this figure are based on predicted binding (as listed on the Los Alamos HIV Immunology Database), all fold changes are represented, regardless of high-binding categorization.

First, we wanted to validate the accuracy of our computational approach in predicting HLA binding of published epitope–HLA pairs reported in the Los Alamos National Laboratory HIV Immunology database (www.hiv.lanl.gov/content/immunology/, accessed 16 February 2022). Published epitope–HLA pairs were computationally evaluated, yielding strong or weak binding predictions in 45.2% of cases (*n* = 204/451). Next, we tested the effects of RAMs on HLA binding for all the peptides that contained a drug resistance-associated site (Figure 1). Notably, HLA-B07:02 exhibited a high frequency of non-permissiveness across multiple RAMs, suggesting that it consistently bound mutant peptides better than wild-type peptides. The highest permissiveness was observed for the HLA-A03:01-K101P, HLA-B15:01-Y143C, and HLA-A02:01-Y188H combinations. Peptides encompassing Y115F (FC: 0.7 to 2.9), Y181I/C/V (FC: 0.03 to 102), M184I/V (FC: 0.7 to 3.5), L76V (FC: 0.3 to 1.2), and R263K (FC: 0.6 to 2.1) bound numerous HLA alleles. The HLA-A24:02-Y181V (FC = 102) and HLA-B39:01-G140A (FC = 89) combinations exhibited the highest FCs in binding, indicating non-permissiveness. In contrast, M46I, G48M/V, I50L, and Q148R were consistently associated with permissiveness across all HLA alleles, a result that agrees with previous work on M46I and G48V [23]. Similarly, the results of B18:01-E138A/G/K showed overall permissiveness, in agreement with results published previously [28,29]. RAMs that are not listed were not previously published in association with specific HLA alleles.

### 3.2. Population-Based Permissiveness of HIV-1 Subtype B RAMs with HLA Alleles Frequent in the African American, European American, and European Populations

**Figure 2:**
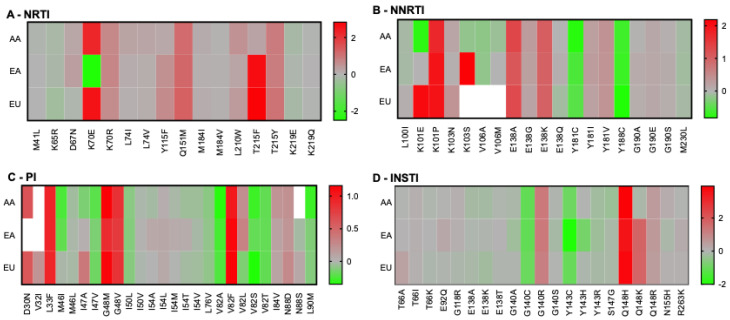
Population-based permissiveness and non-permissiveness heatmaps of HIV-1 subtype B RAMs in the AA, EA, and EU populations. Average log-transformed fold changes in binding are color-coded. Red indicates non-permissiveness, green permissiveness, and grey neutrality. White indicates no binding. Peptides that were neither weak nor strong binders were excluded from downstream analyses, explaining why not all RAMs are represented in this figure.

Given the overall consistency between our predictions and published results [23,28,29], we reflected that possible biases in the selection of drug resistance mutations caused by specific HLA alleles may be visible at the population level, as reported by others [28,29,30]. Considering that antiretroviral drugs are generally available to people in Europe and the USA, we selected the most frequent HLA alleles in the African American (AA), European American (EA), and European (EU) populations for further analyses. As mentioned in the Section 2, only 9-mer peptides were used for this analysis, all derived from subtype B, because it is the most common subtype in Europe and the USA. Some alleles are frequent in all populations, whereas others are only found above 5% in a specific group (Table 2). For example, HLA-A02:01, HLA-B35:01, and HLA-C04:01 are prevalent in all populations, while HLA-A30:01 is only found to be >5% in African Americans. Our analyses permitted the calculation of overall permissiveness and non-permissiveness of bound RAMs in each population (Figure 2). This analysis was performed on a number of peptides different from those in Figure 1, because peptides that were neither weak nor strong binders were excluded from downstream analyses. Our results generally showed comprehensive similarities in permissiveness and non-permissiveness between the three populations. Notable exceptions included the overall permissiveness of K70E in the EA population (FC = 0.003) but non-permissiveness in the AA or EU populations (FC = 177 and 370, respectively). K101E was associated with permissiveness in the AA population but with non-permissiveness in the EU population. Some RAMs did not significantly bind any HLA alleles in specific populations: K103S and V106A/M in the EU population; D30N and V32I in the EA population; and V32I and N88S in the AA population. For the EU population, K103S did not bind, while K103N was associated with mild non-permissiveness. This was due to the absence of the binding of the WT peptide, whereas K103N, but not K103S, was a predicted binder from which an FC could be calculated. Notably, this observation is compatible with previously published T-cell antigenicity of a K103N containing 9-mer but not its WT counterpart [27].

### 3.3. RAMs in HIV-1 Subtype C and HLA Permissiveness in the South African Population

**Figure 3:**
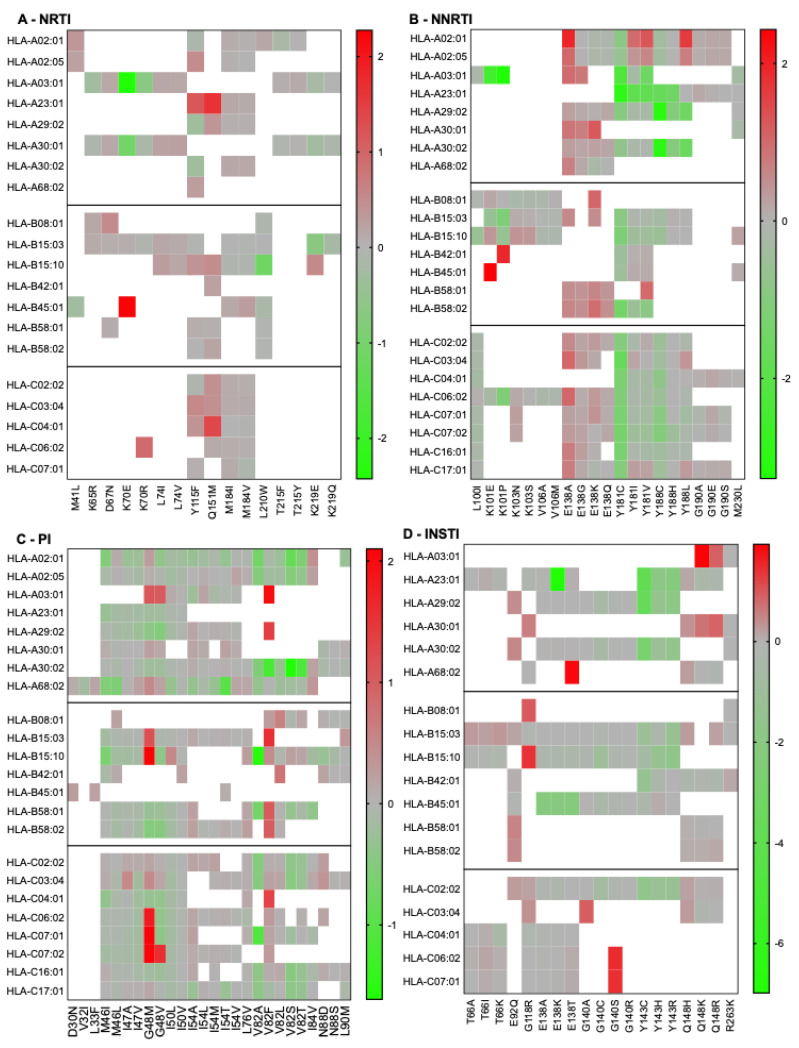
South-African population-based HIV-1 subtype C RAM permissiveness and non-permissiveness heatmaps. Average log-transformed fold changes in binding are color-coded. Red indicates non-permissiveness, green permissiveness, and grey neutrality. White indicates no binding. Peptides that were neither weak nor strong binders were excluded from downstream analyses, explaining why not all RAMs are represented in this figure.

Given that a large number of people with HIV live in South Africa, where treatment is frequently available, and the dominant HIV-1 subtype is C, we performed similar analyses with this subtype and HLA alleles frequently found in the South African population [41]. Figure 3 illustrates the heatmaps of RAMs/HLA permissiveness.

**Figure 4:**
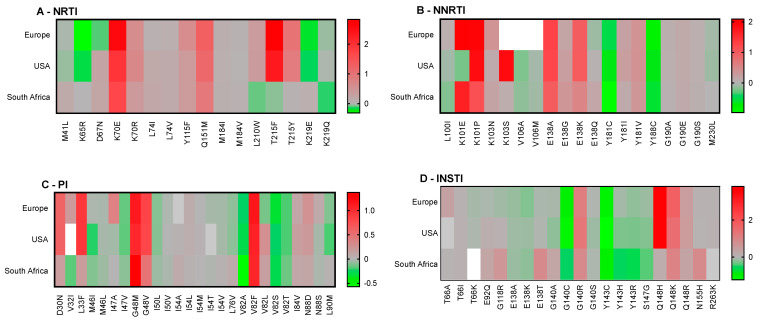
Comparison of population-based permissiveness and non-permissiveness heatmaps between Europe, the USA, and South Africa. Average log-transformed fold changes in binding are color-coded. Red indicates non-permissiveness, green permissiveness, and grey neutrality. White indicates no binding. Peptides that were neither weak nor strong binders were excluded from downstream analyses, explaining why not all RAMs are represented in this figure.

Notable observations include the general non-permissiveness of RT-E138 substitutions and the large contribution of HLA-C alleles to RAM permissiveness. We noted that HLA-B15:03 bound most of the peptides tested, suggesting low specificity of this allele. Next, we combined our population-based analyses to compare predicted permissiveness between different regions (Figure 4). This analysis showed that certain RAMs, such as K65R or V32I, may be associated with permissiveness in specific populations. In contrast, other RAMs (e.g., T215Y, K101E, Q148H) were associated with non-permissiveness exclusively in certain populations.

### 3.4. Permissiveness and Non-Permissiveness in the Context of HLA-B57:01

**Figure 5:**
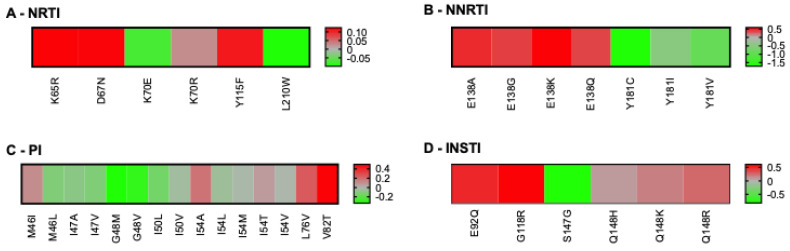
Heatmaps of RAMs/HLA-B57:01 permissiveness with HIV-1 subtype B. Log-transformed fold changes in binding are color-coded. Red indicates non-permissiveness, green permissiveness, and grey neutrality. RAMs that were neither weak nor strong binders of HLA-B57:01 are not represented in this figure, for example L74V and M184I/V. All RAMs were used for this analysis (none excluded), because no RAM is exclusively associated with ABC, which is itself not recommended for people with HLA-B57:01. Similarly, no drug class was excluded from this analysis, as HLA-B57:01-positive persons are eligible to receive any treatment combination, as long as it does not include ABC.

Although our population-based analysis provided several testable observations that could be validated by using epidemiological data that were not available to us, we also considered that we had access to some specific data. People with HIV must be tested for the HLA-B57:01 allele before using abacavir, because carriers are at an increased risk of hypersensitivity reaction to this drug [49]. Accordingly, HLA-B57:01 is the most frequently documented allele for people with HIV. We thus examined which RAMs were predicted to be associated with permissiveness vs. non-permissiveness under HLA-B57:01 (Figure 5). In the category of RAMs against the NNRTI drug class, our results indicated that Y181C/I/V was associated with strong permissiveness, whereas RAMs involving RT-E138 were associated with non-permissiveness. S147G was the only integrase RAM associated with permissiveness; the others were generally linked to non-permissiveness. Common RAMs that are not listed in Figure 5 were not predicted to significantly bind HLA-B57:01.

The availability of linked HLA-B57:01 screening results and HIV resistance genotypes from the British Columbia Centre for Excellence in HIV/AIDS provided an opportunity to compare our computational predictions with clinical data. This dataset included 2320 participants with HIV-1 subtype B who had undergone HLA typing and HIV protease/RT genotyping and had consented to their data being used for HIV research. Of these, 941 individuals had also undergone HIV integrase genotyping. Of all the above RAMs, only the Y181C substitution differed significantly in its distribution between HLA-B57:01-positive and -negative people. Specifically, Y181C was found twice among 168 HLA-B57:01-positive (1.21%) versus 153 times among 2152 people without HLA-B57:01 (6.1%), yielding an odds ratio of 0.16 (*p*-value = 0.001, q-value = 0.04), indicating that Y181C is significantly under-represented among HLA-B57:01-expressing persons. Somewhat surprisingly, this result initially appeared contrary to our computational predictions that suggested permissiveness (FC = 0.1) for HLA-B57:01-Y181C (Figure 5B).

Given this apparent discrepancy, we further investigated circumstances under which Y181C peptides may better bind HLA-B57:01 than their WT counterparts. To do this, we performed analyses using 9-mers to 11-mers spanning position 181, because HLA-B57 can bind peptides in this length range [50]. Although these extended analyses confirmed that, overall, Y181C decreased HLA-B57 binding, we observed that Y181C increased HLA-B57 binding specifically when the Y/C residue was at the major anchor position P2 for 9- to 11-mer peptides (“non-permissiveness”, Table 3). By contrast, when residue 181 lay at another position, including the C-terminal anchor PΩ (P9), Y181C reduced HLA-B57:01 binding (permissiveness). Importantly, it has been reported that HLA-B57 binding is disfavored by the presence of a tyrosine (Y) at P2 compared to a cysteine (C) [50]. Thus, in certain circumstances, the computational predictions can be reconciled with clinical observations.

However, because anchor usage varies across different HLA alleles, we did not repeat all analyses using RAMs at P2 as a constraint. In addition, our cohort analysis did not control for treatment history (i.e., the analysis was not restricted to participants who were receiving, or had previously received, NNRTIs) to include potential transmitted drug resistance mutations in addition to the acquired resistance mutations. Also, given the protective nature of HLA-B57, it is possible that people expressing this allele could be less likely to experience virological failure.

## 4. Discussion

The interplay between immune escape mechanisms and RAMs has profound implications for both HIV evolution and clinical outcomes. For instance, RAMs in the viral protease (PR) gene, including the M46I, I47A, and G48V substitutions, can impair the CD8+ T-cell response [23]. Similarly, HLA-B40:02-restricted CTLs effectively targeted cells presenting wild-type (WT) integrase (IN) peptides but exhibited diminished activity against cells presenting the E92Q substituted peptide [24]. E92Q causes a reduced susceptibility to the integrase strand transfer inhibitors raltegravir and elvitegravir. In another case, CTLs from one patient were able to recognize the lamivudine-resistant M184V peptide presented by HLA-A2 [25]. HLA-B27 has been associated with the enhanced binding of K65R mutant peptides [26]. Another study reported the CTL recognition of an epitope containing the K103N substitution in reverse transcriptase but not its WT counterpart [27]. M184V, K65R, and K103N are common RAMs against nucleoside, nucleotide, and non-nucleoside reverse transcriptase inhibitors, respectively, which are very clinically relevant.

The influence of HLA alleles on HIV evolution is also evident at the population level. For example, the E138G/A/K RAMs in reverse transcriptase (RT), which confer resistance to rilpivirine, also decrease HLA-B18-restricted CTLs’ responsiveness and were more prevalent in people expressing HLA-B18 than people without this allele [28]. This observation was supported by a study of the Swiss HIV cohort, which confirmed that individuals expressing HLA-B18 had a higher risk of developing E138 RAMs, while HLA-B35 carriers were more likely to develop the V179 substitution [29]. In a Kenyan cohort, individuals with the HLA-A*66:01 allele demonstrated an increased risk of acquiring the T97A integrase RAM [30]. T97A is clinically relevant, because it can significantly increase resistance levels associated with other integrase RAMs, essentially conferring resistance to the entire integrase strand–transfer inhibitor drug class [31]. Circulating HIV strains can also exhibit pre-adaptation to common HLA alleles within a population. Such pre-adapted viruses can accelerate disease progression in individuals who acquire them [32,33]. This phenomenon may also influence ART outcomes. Specific HLA adaptations, such as B∗15:01-I93L in protease and B∗51:01-I135T, B∗07:02/05-S162C, B∗35:03-E297A, B∗15:01-Q334L, and A∗32:01-E399D in reverse transcriptase, are prevalent in some regions with high levels of viral pre-adaptation to these alleles [34].

These insights underscore the complex and dynamic relationship between immune pressure, drug resistance, and viral evolution, with potential implications for both therapeutic strategies and population-level disease management.

In this study, we used computational predictions to assess how RAMs affect the binding of HIV peptides to HLA. Our results showed that certain RAMs associated with either permissiveness or non-permissiveness, often in agreement with previously published work [23,27,28,29]. The changes in the HLA allele binding capacity induced by RAMs were calculated from binding activities of predicted weak and strong binding peptides. The exact mechanisms associated with binding changes from RAMs are not completely understood.

We also identified RAMs that may be selected more frequently in specific populations based on HLA allele frequencies, generating several testable hypotheses. For example, African Americans may be more likely to exhibit the Q148K mutation than European Americans. Conversely, African Americans and Europeans may be less likely to acquire the K70E substitution than European Americans. The latter may, in turn, have reduced the prevalence of the K103S substitution compared to other groups. Data from large insurers in the USA could help confirm these predictions at a clinical level.

Our analyses yielded predictions that could also be tested in global epidemiological dataset, assuming comparable ART regimens and other clinical parameters. Given the global use of dolutegravir, testing the hypothesis that N155H may be less frequent in South Africa than Europe or the USA would be particularly interesting. Although less frequent than R263K or G118R, the signature mutations for dolutegravir [51,52,53,54], N155H can emerge upon failure with this drug [55]. Importantly, we note that comparing different subtypes adds an additional confounding factor that is the subtype-specific effects of RAMs on viral fitness, such as those published for R263K in subtypes B and C [56]. This parameter does not apply to the European and American populations that are both affected mainly by subtype B. While we examined several HLA alleles and mutations, expanding this analysis to include other HLA loci and broader population groups (e.g., in Asia or Latin America) would enhance the generalizability of our findings.

Despite these interesting results, our study has several limitations. First, population-level predictions were performed on individual alleles, whereas people typically express multiple alleles simultaneously. The combined effect of HLA alleles with contradictory outcomes in regard to permissiveness cannot be assessed with our current tools. This issue is best illustrated by the following apparent contradiction: K70E is commonly found in treatment-experienced persons in South Africa [57], whereas our analysis indicates this mutation to be overall non-permissive (Figure 4). However, further examination indicates that, although K70E was about 2-fold non-permissive for HLA-B45:01, it was also 0.8 to 2-fold permissive in respect to HLAA03:01 and 30:01 (Figure 3). For the same reason, we could not account for linkage disequilibrium between specific HLA alleles. Second, and related to the first point, we did not use HLA frequencies in specific populations to weigh the effects of RAMs on permissiveness. Third, our analyses did not take into consideration the position of the RAM within the peptide; although, we assumed that NetMHC considered this parameter in its predictions. Instead, we averaged out results for all positions. Further analyses may explore ways to address this limitation, e.g., weigh the peptides with RAMs at specific positions. Fourth, the data that were used to select HLA alleles for each population (African American, European, and European American) under-represented the EA and AA populations by 10 and 40 times, respectively (Table 2). Most importantly, we were unable to draw conclusions about the clinical applicability of our computational predictions.

Indeed, we report here, for the first time, the under-representation of the Y181C substitution in people expressing HLA-B57. Although several confounders may have influenced this clinical observation, our overall (not limiting Y181C to P2) predictions conflicted with the clinical data. Similar analyses on HLA-B57 and Y181C with another prediction tool called MHCseqNet as well as NetMHCpan v.4.1 yielded comparable results [45]. Nevertheless, it is notable that the clinical data did align with HLA binding predictions when Y181C was only at the anchor position P2. Given the critical importance of P2 in HLA-B57 binding [50], this outcome is unlikely to be coincidental and may highlight a potential shortcoming of current prediction models.

Accordingly, our population-based predictions must be considered with caution until the hypotheses we have proposed are tested. Quantifying the interplay between multiple HLA alleles and their evolutionary pressures remain complex. In the future, large language models with advanced computational capabilities may improve prediction accuracy and our ability to integrate the effects of several alleles concomitantly, provided sufficient experimental data are available for training. Overall, our study lays out a conceptual framework to study the clinical and epidemiological implications of HLA alleles on the emergence of HIV RAMs at the individual and population levels. The principles exposed in this paper could in theory apply to other pathogens and pathologies associated with genetic changes, such as cancer. As such, our study also advocates for the integration of HLA typing into research on existing clinical datasets and cohorts.

## Figures and Tables

**Table 1:** List of major drug resistance-associated substitutions against different drug classes used in this study, as defined by the Stanford HIV Drug Resistance Database (v.9.2).

Drug Class	RAMs
Integrase strand transfer inhibitors	T66A, T66I, T66K, E92Q, G118R, E138A, E138K, E138T, G140A, G140C, G140S, G140R, Y143C, Y143H, Y143R, S147G, Q148H, Q148K, Q148R, N155H, R263K
Nucleoside and nucleotide reverse transcriptase inhibitors	M41L, K65R, D67N, K70E, K70R, L74I, L74V, Y115F, Q151M, M184I, M184V, L210W, T215F, T215Y, K219E, K219Q
Non-nucleoside reverse transcriptase inhibitors	L100I, K101E, K101P, K103N, K103S, V106A, V106M, E138A, E138G, E138Q, E138K, Y181C, Y181V, Y188L, G190A, G190S, G190E, M230L
Protease inhibitors	D30N, V32I, L33F, M46I, M46L, I47A, I47V, G48M, G48V, I50L, I50V, I54A, I54L, I54M, I54T, I54V, L67V, V82A, V82F, V82L, V82T, V82S, I84V, N88D, N88S, L90M

**Table 2:** Rates of the most frequent (>5%) HLA alleles in the European (*n* = 21,571), European American (*n* = 2248), and African American (*n* = 564) populations used in this study [38,39,40].

HLA Allele	European	European American	African American
HLA-A01:01	10–15%	16%	
HLA-A02:01	>20%	26%	12%
HLA-A03:01	10–15%	13%	9%
HLA-A11:01		7%	
HLA-A24:02	10–15%	8%	
HLA-A30:01			8%
HLA-A30:02			6%
HLA-A33:03			5%
HLA-A74:01			5%
HLA-B07:02		13%	7%
HLA-B08:01		11%	
HLA-B15:01		6%	
HLA-B35:01	>20%	6%	7%
HLA-B35:03	>20%		
HLA-B42:01			6%
HLA-B44:02	>20%	7%	
HLA-B44:03			5%
HLA-B51:01	>20%	5%	
HLA-C02:10			6%
HLA-C03:03		5%	
HLA-C03:04		7%	
HLA-C04:01	>10%	7%	20%
HLA-C05:01		7%	
HLA-C06:02		7%	9%
HLA-C07:01	20–40%	14%	12%
HLA-C07:02	20–40%	12%	7%
HLA-C07:06	20–40%		
HLA-C12:03		6%	
HLA-C16:01			9%
HLA-C17:01			8%

Blank cells indicate an allele frequency below 5%.

**Table 3:** Binding affinities and fold changes in HLA-B57:01 binding of 9-, 10-, and 11-mers spanning position 181. Increased RAM binding (FC > 1) is indicated in bold and underlined.

	WT	Y181C	Affinity WT (nM)	Affinity Y181C (nM)	FC
9-mer	KQNPDIVIY	KQNPDIVIC	10,541.8	15,082.7	0.70
	QNPDIVIYQ	QNPDIVICQ	32,259.5	34,363.3	0.94
	NPDIVIYQY	NPDIVICQY	23,451.7	24,244.5	0.97
	PDIVIYQYM	PDIVICQYM	29,611.2	33,195.3	0.89
	DIVIYQYMD	DIVICQYMD	33,387.3	35,307.7	0.95
	IVIYQYMDD	IVICQYMDD	25,116	25,813.3	0.97
	VIYQYMDDL	VICQYMDDL	22,674.4	28,647.8	0.79
	**IYQYMDDLY**	**ICQYMDDLY**	**26,921.8**	**20,628.5**	**1.31**
	YQYMDDLYV	CQYMDDLYV	24,214.4	25,038.2	0.97
10-mer	RKQNPDIVIY	RKQNPDIVIC	14,073.5	17,708.8	0.79
	KQNPDIVIYQ	KQNPDIVICQ	18,394.2	21,798.1	0.84
	QNPDIVIYQY	QNPDIVICQY	26,064.5	28,065.1	0.93
	NPDIVIYQYM	NPDIVICQYM	23,506.3	25,071.8	0.94
	PDIVIYQYMD	PDIVICQYMD	34,629.8	36,405.6	0.95
	DIVIYQYMDD	DIVICQYMDD	34,060.8	34,366.6	0.99
	IVIYQYMDDL	IVICQYMDDL	21,105.3	22,379.3	0.94
	VIYQYMDDLY	VICQYMDDLY	18,940.7	27,438.2	0.69
	**IYQYMDDLYV**	**ICQYMDDLYV**	**25,849.7**	**23,556.9**	**1.10**
	YQYMDDLYVG	CQYMDDLYVG	23,955.3	24,907.9	0.96
11-mer	FRKQNPDIVIY	FRKQNPDIVIC	28,212.5	30,168.4	0.94
	RKQNPDIVIYQ	RKQNPDIVICQ	29,171	31,073.8	0.94
	KQNPDIVIYQY	KQNPDIVICQY	17,142.8	21,485.2	0.80
	QNPDIVIYQYM	QNPDIVICQYM	33,385.1	35,156.4	0.95
	NPDIVIYQYMD	NPDIVICQYMD	35,814.5	35,965.5	1.00
	PDIVIYQYMDD	PDIVICQYMDD	40,804.6	41,305.7	0.99
	DIVIYQYMDDL	DIVICQYMDDL	35,541.9	36,375.3	0.98
	IVIYQYMDDLY	IVICQYMDDLY	24,685.2	25,753.6	0.96
	VIYQYMDDLYV	VICQYMDDLYV	33,265.4	35,260.4	0.94
	**IYQYMDDLYVG**	**ICQYMDDLYVG**	**34,494**	**30,751.7**	**1.12**
	YQYMDDLYVGS	CQYMDDLYVGS	35,590.4	36,039.9	0.99

## Data Availability

Raw data will be made available upon request.
